# Abstract Proceedings Buffalo Medical Association

**Published:** 1878-09

**Authors:** L. Howe

**Affiliations:** Sec’y, pro tem.


*ART. II.—Abstract of the Proceedings of the Buffalo Medical Association,* Aug. 6th, 1878.

The President, Dr. Thomas Lothrop in the Chair: Present, Drs. Rochester, Cronyn, Samo, Bartlett, W. W. Miner, Barnes, Brecht, J. F. Miner, Fowler, Howe, and by invitation Dr. Hartwig.

The minutes of the last meeting having been read and approved, Drs. A. M. Barker and Mary B. Moody were elected members. Dr. Bartlett, the essayist for the evening, was called upon and read the following paper on Accidental Hemorrhage.

TREATMENT OF ACCIDENTAL HEMORRHAGE.

*Mr. President and Gentlemen of the Association:*

The brief time allowed me to prepare my paper has prevented my making extensive investigations into the literature of the past regarding the ancient treatment of accidental hemorrhage; and, your professional reading has made you familiar with much which I gladly omit. I shall seek the virtue of brevity and draw mainly upon my own experience in the treatment of such accidents.

I suppose it is expected that members who offer papers here, particularly those whose experience covers a score or more of years, will rehearse the special treatment by which they have succeeded in difficult and exceptional cases. We owe this to the young, aspiring company which has joined us in the last few years. We should tell of our difficulties and the means by which we surmounted them, when all our previous knowledge, and all the wisdom of the books failed to aid us.

I shall confine my remarks to hemorrhagic misfortunes which precede, accompany, or follow physiological or pathological changes, and first of all refer to Epistaxis.

Nose-bleed is, perhaps, the most common of all hemorrhages, and often proceeds from trifling causes, especially in those of hemorrhagic diathesis. Not unfrequently, it is positively alarming from its copiousness and the failure of attempts at suppression. Occurring in the course of certain phlegmasiæ, as for instance, typhoid fever, it may decide the case adversely to the patient. It is to a certain extent, perhaps, salutary in some cases, but excessive losses are always detrimental.

The means usually recommended and employed are cold, styptics, as alum, salts of iron, lead, copper; position of head, packing of the nostrils with cotton or other fibrous material, with or without styptic solutions; plugging of the posterior nares by similar materials drawn into place by a flexible catheter or the instrument of Bellocq; external pressure in divers irregular ways; the elevation of hands above the head; and, finally by astringents addressed to the general circulation. In spite of these varied means, death, not unfrequently follows epistaxis, either by sudden collapse or the influence exerted upon the course of existing disease.

The means I have found singly and combined to be an effectual remedy for epistaxis is pressure external or internal. The experience of more than twenty years has convinced me that the ordinary forms of styptic treatment—or packing as ordinarily practiced are wholly unreliable in cases at all severe, or appearing in the course of disease.

In June 1863, I had charge of a case of typhoid fever occurring in a phthisical young gentleman residing on Seneca street, near Michigan. The disease was protracted and the patient became exceedingly feeble from diarrhea and loss of digestive power. Early one morning, I was called to find him bleeding from the nose to a most alarming extent. I used styptics, plugged the anterior nares, and with the assistance of an eminent physician, now deceased, tightly closed the posterior nares—all these proceedings failed to do more than check,—they did not stop the hemorrhage. In certain positions the loss, drop by drop, passed outward, in others inward. General treatment had no perceptible effect, and after 36 hours perseverance I found my patient rapidly failing. In this dilemma I removed all the packing from the nares, when the slow drop, drop, passed outward. At last by pressure applied to the angular branch of the external carotid, I had the satisfaction of finding the hemorrhage fully controlled. This I accomplished by application of the thumb and finger at the lower border of the Ossa Nasi externally. Temporary removal of pressure was followed by hemorrhage, and to secure continuous pressure, I shaped the jaws of a spring clothes pin to fit the nose, removing a portion of the wire spring to lessen pressure. This simple tourniquet remained in place and the patient recovered. Satisfied, by this experience, of the doubtful utility of packing in the usual way, I felt quite safe until a case of nasal hemorrhage occurred in a patient about 14 years old, residing on Scott street, near Washington. The lad had suffered previously, and his mother did not feel alarmed until he fainted from loss of blood. Before my arrival he slightly rallied only to sink exhausted by fresh bleeding. He was pulseless, sightless, breathless, and to all appearance dead. I forced some whiskey down his throat and practiced artificial respiration. As he gasped, I passed a sponge tent, intended for other use, into the right nostril from which I supposed the blood had flowed. It soon expanded and filled the nostril posteriorly and anteriorly, completely controlling the hemorrhage I left the tent in position forty-eight hours; on removal, there was no loss of blood, and I had the pleasure of being assured years afterwards that the treatment had been radically successful. Since that date, August 1863, I have used the sponge tent in all alarming cases of hemorrhage of the nose. My cases number 27, comprising those occurring in Typhoid, Diphtheria, Scarlatina, traumatic injuries and causes not capable of tabulation. I think I may fairly claim originality in this proceeding.

Another form of accidental hemorrhage is that known as Alveolar, following the extraction of teeth. Instances of death are quite frequent from this cause. I recall a case in which I used a treatment somewhat novel. The patient was a soldier, discharged for sickness and on his way to friends at the West. He had suffered from acute hepatitis and had excessive ptyalism, in which condition a tooth was extracted by a dentist. When seen by me, he had bled apparently five or six pints, and the blood was passing in a fine needle-like stream from the inferior maxillary. Every effort to arrest it had been made, apparently, by styptics, etc, and still the hemorrhage went on. Finding plugging the cavity of no avail, I procured a large cork and hollowed it so as to clasp the lower edge of the inferior maxillary, just back of the dental foramen, to compress the inferior dental. Pressure was then made by a band carried over the head, a small cork tapered to pass well down upon the bottom of the cavity, the jaws closed so as to make pressure upon it and the band tightened to make pressure upon the dental artery externally. Hemorrhage ceased and did not return. I recommend plugging the cavity left by the removed tooth, with a slender cork, which has first been conveniently spitted upon an ordinary dining fork. This is very convenient for keeping the cork in place, and the patient is advised to keep the jaws firmly compressed.

Hemoptysis is another form of hemorrhage which is frequently exceedingly difficult to control, and, for which numberless remedies have been proposed. It has been my lot to attend a very large number of such cases and watch the effect of many remedies.

For ten years past, I have used the following formula.

℞ Tr. Digitalis, . . ʒis,

Ol. Terebinth, . . ʒiij,

Ol. Menth. Pip. . gtt–x,

Acidi Sulph. Ar. . ʒiij,

Alcoholis, . . . q. s.

Ad fac. . . . . ℥ij. M.

Dose 40 to 60 drops well mixed with sugar, to which one or more tablespoonfuls of water may be added, every two, three or four hours, according to urgency of the hemorrhage. I know of no combination that all approaches it in efficiency. Acids,—Tannic, Gallic, Sulphuric,—Turpentine, Ergot, Lead Acetate with or without opium, are all greatly inferior in their effects.

In Hematemesis, Tr. Ferri Chloridi has proved the most efficient remedy. In Hematuria, Tr. Benzoin Co. and Tannic Acid. I have never attributed any special utility to Ergot as a haemostatic, except in certain forms of Uterine Hemorrhage. In the form of Ergotine it may be used hypodermically with advantage, but the Tincture and Extract are very unreliable. It is very often rejected by the stomach, particularly if combined with opium or sulphuric acid.

Umbilical hemorrhage, or bleeding from the divided umbilical cord, is a very frequent misfortune and occurs when apparently every precaution has been taken by careful ligation. Many infants die from this cause, and many are rescued by the merest accident. I have found the plan of enclosing a small segment of the cord in a second loop of the ligature, a perfect safeguard. It compels the formation of a clot of blood between the two points of ligation, and if carefully done may always be trusted.

It is a trifle difficult to draw the line between accidental and unavoidable uterine hemorrhage. The position of the placenta when centrally implanted is itself an accident, and hemorrhage, in such circumstances should be classed as accidental.

No more important subject could occupy the hour, than the consideration of Uterine Hemorrhage. To its observation the best minds of our profession have given earnest and continuous thought. Numerous are the plans, remedies, and devices for restraining these hemorrhages, and fortunately now-a-days in a very large number of cases some one of them succeeds. Hot water injected into the uterus has been highly praised in post partum hemorrhage from whatever cause. I think it has sound philosophy in its favor. The practice of introduction of astringents notably Sol. Ferri, Per-Sulph. by injection, has high professional sanction, and, yet seems hardly devoid of danger. We need further experience before deciding affirmatively in its favor.

June 28th, 1871, I was summoned in haste to attend my patient, Mrs. D., residing upon Carroll street, near Chicago. On my arrival, I found Dr. A. H. Briggs had also been called and he kindly consented to remain and assist me in the conduct of the case. Examination showed a foot presentation, but I was struck

ARMY MEDICAL LIBRARY WASHINGTON

by the extraordinary œdema of the patient; particularly of the lower extremities. The feet were enormous transparent sacks of water, and the whole body anasarcous. In an hour, unaided by special art, a child was born, and it was then evident that there was twin pregnancy. The hemorrhage was only ordinary until birth of second child when it became suddenly profuse and the weak anæmic patient was rapidly prostrated. On attempting to deliver the placenta, I found true hour-glass contration, the double placenta tightly compressed in the upper segment and the hemorrhage absolutely appalling. While I proceeded to dilate the constriction, Dr. Briggs, at my request, hastened to the nearest drug-store and obtained two sponges of the size of an ordinary lemon, and, a bottle of Sol. Ferri per Sulph. On his return the patient was unconscious and pulseless, and the hemorrhage still continued though placenta was removed. Hastily attaching a piece of tape to a sponge, I saturated it with diluted Sol. Ferri per Sulph. and carried it to the fundus of the womb. The hemorrhage ceased instantly, the patient to all appearance dead, slowly rallied and made a good recovery. Four hours later, I withdrew the sponge, a hard stony mass, and, the womb contracted well. I can find no record of a similar proceeding, but it seems to me to be preferable to the mere injection of astringents. The Solut. Fer. per Sulph. coagulated the blood and the sponge formed a tampon upon which the womb could and did contract.

In the treatment of abortions and premature deliveries, the principal difficulty lies in the safe management of hemorrhages. I am not a convert to the specific effect of ergot in such cases. I believe that in these, as in most hemorrhages, we need pressure upon the bleeding vessels. Mere styptic effects are not to be trusted.

The Tampon is a good illustration of our philosophy in this matter, but the tampon will often partially or wholly fail. There is danger in forcing loose fibrous or other material into the cavity of the womb, not simply by the force used, but also from fragments retained producing uterine irritation, leucorrheal discharges, etc. In such cases when unable to reach and remove the placental substance and membranes, I am accustomed to pass a sponge tent by the speculum, and then tampon in the ordinary way, upon it. Generally, I find, upon withdrawing the tampon, the dilated sponge tent follows, dragging with it the rudimentary fragment of the placenta. The hemorrhage is also very much less than when the ordinary tampon only is used.

In accidental hemorrhage from supposed partial detachment of the placenta at or near term, I am inclined to favor the induction of labor and of artificial delivery as soon as dilation of the cervix will permit. The well known diagnostic distinction between it and placenta prævia should be borne in mind, viz: in partial non-cervical detachment, hemorrhage is most marked between pains; in placenta prævia at time of pain. The diagnosis is thus made easy. So soon as forceps can be applied the delivery should be accomplished. Delays are infinitely dangerous in such cases. I have usually ruptured the membranes early so as to obtain the aid of the fœtus as a tampon, and I usually also apply a broad bandage firmly over the abdomen, interposing a liberal layer of cotton batting to equalize pressure. I think if we have any reasonable theory in such cases it must be that of compression; the grand fundamental idea in all cases where applicable. Turning may be useful but its risks to the mother should be considered.

In the treatment of certain forms of hemorrhage, particularly such as occur at or near the so called "Change of life," I place great confidence in carbolized sponge tents introduced within the cavity of the uterus. I have never seen ill effects and have frequently controlled, by a single tent, hemorrhages which had resisted the ordinary tampon and various astringents. I introduce the tent through the speculum, well up to the fundus, and tampon over it in the usual way. A recent writer reports that he frequently removes small polypi by this means.

Accidental hemorrhage may also occur from ulceration of the os uteri or cervical canal. These I treat by applications of Cupri Sulph. in form of pencils, touching not only the bleeding ulcers, but also the cervical canal. Sulph. Copper is also very useful in simple abrasion or congestion of the cervix.

Polypi are removable by traction, evulsion and the ligature. A

case occurring in my own practice illustrates the necessity of careful scrutiny into causes of accidental hemorrhage.

On making an obstetrical engagement I was informed that the lady had lost her child at a previous labor and endangered her own life by hemorrhage, and special care was desired from this misfortune. Summoned to attendance on the labor, I found that dangerous hemorrhage had already occurred. The child was soon born, exceedingly exsanguine, and lived but twelve hours. There was good contraction of the womb and I used all required care, remaining four hours with the patient with no recurrence of hemorrhage. An hour later, I was called to find my patient almost pulseless from flooding. Examination showed the os open and dilatable and I passed with little effort my fingers within it, when they came in contact with a pendulous body which I recognized as a polypus. It was the size of a small peach, and fortunately had a slender pedicle. I removed it by torsion without difficulty. There was no further hemorrhage, and two confinements since have been without unusual hemorrhage, and safe to both mother and children.

The subject of accidental hemorrhage is but slightly considered in the remarks I have made, but longer trespass upon your indulgence would be tedious, and special points can be brought up by discussion. I have offered personal experience, a course I commend particularly to all whose experience has been considerable, and as the only one tending to the accumulation of Medical facts. There is value in carefully prepared statistics and generalizations, but infinitely more in the presentation of special cases and carefully observed phenomena.

It is not the splendid operation only, but the modest success that practically aids in our daily work.

This paper was prepared for our last meeting but I was unavoidably absent. Since that date I have treated a case of epistaxis in a lad of ten years, a son of Mr. Flynn, Cor. Scott and Red Jacket streets. By repeated bleedings the boy was exceedingly exsanguine. A tent was inserted in the right nostril and left for nearly three days. No hemorrhage. On withdrawal a few drops of blood passed, but the lad has had no return in the last three weeks.

Post partum hemorrhage, though occasionally accidental, is usually avoidable. Position, and more particularly, *deference* to nature's mode of placental expulsion would largely diminish the dangers encountered. The reprehensible practice of delivering the Placenta by traction upon the cord cannot be too severely condemned. I have for years practiced what is now known as Crede's method, and always bring the placenta into the vagina by external manipulation; traction upon the cord is then useful and justifiable. The rude separation of the placenta from the uncontracted uterine wall is one of the most frequent causes of undue and dangerous flooding.

I am compelled to close my remarks without reference to many forms of hemorrhage, properly called accidental, but as so far as I know my practice accords with that of *others,* it is somewhat foreign to my purpose to review them. I have aimed to offer you my personal experience which is in some respects unique. The use of sponge tents for epistaxis, dating back no less than eighteen years is original with myself so far as I can learn. Two English Physicians in '74, I think, allude to their use; but no work, unless it be very recent, makes any allusion to them for this purpose. I can recommend them as truly specific in such cases. Their size should vary as also their length. They should always pass well to the posterior nares, and be left from forty-eight to seventy-two hours in position; contrary to supposition the withdrawal is quite painless. The error, if any, will be in using tents of too great diameter. I have never used one of more than three lines average diameter, for children two lines is ample. The important consideration is their length. This should be two and one-half to three inches for an adult, and two inches for a child from six to ten years. The tent should be slightly curved and passed well back by the middle meatus of the nostril from which the blood is supposed to flow. No part of the tent should project anteriorly from the nostril, and the usual traction loop should be shortened so as to be out of way. This will lessen danger of interference by the the patient. The removal should be accomplished by rotation and traction. I have never noticed displacement of the Vomer or injury to the turbinated bones; a slight loss of blood is to be expected, but it soon ceases.

With thanks for your kind attention I invite your friendly criticism.

Dr. W. W. MINER was afraid that sponge tents might produce injury to the turbinated bones, when used to pack the nares.

Dr. ROCHESTER believed that we had very efficient means of controlling many such hemorrhage in the hypodermic use of ergot. He was free to say however, that a great objection to its employment was its tendency to produce local abscesses, or even sloughs. On one occasion he was obliged to treat an arm thus affected for nearly six months, and had seen the same accidents occur in the practice of other physicians. When administered internally, he considered ergot of little use.

Dr. CRONYN thought that the essayist had, to a certain extent, confused the classification, by regarding accidental and unavoidable hemorrhage as the same. The use of tents of dry sponge in epistaxis was as old he believed as Bellocq's canula. In general, however he agreed with the writer and would thank him for presenting the subject so acceptably. One other method he often used for arresting epistaxis, which consisted simply in folding the hands above the head. A rational explanation of the manner in which this produced the desired effect, had been offered some years before.

As to giving ergot by the stomach he considered it useless, but hypodermically it is of great value in arresting many of the accidental hemorrhages. He thought the abscesses were often due to the occasional introduction of the needle into the muscular tissue.

Dr. HARTWIG had seen such results as those mentioned by Dr. Rochester, and considered them to be due to the idiosyncracy of the patient. He could hardly conceive how an intelligent physician could introduce the needle into muscular tissue.

Dr. BARTLETT replied to these criticisms by saying that the use of tents of compressed sponge was original with him as far as he was aware, and did not remember having seen it employed by others. The means proposed were only such as he would use when prompt action was demanded, and when resort to medication of any kind would necessitate some delay.

Under the head of Voluntary Communications, a number of interesting pathological specimens were presented—as follows.

Dr. J. F. MINER presented specimens of Sub-Mucous Uterine fibroids recently removed. They gave rise to menorrhagia or perhaps metrorrhagia,—continuous hemorrhage greatly aggravated at the menstrual period. Perhaps Dr. Bartlett would include it under the head of accidental hemorrhage, though it seemed to be upon a well considered and uniform plan of nature in such cases.

He also presented a Capsular Cataract, recently extracted from the eye of a lady, who twenty years since had been operated upon by the then common method of *depression*. It was a rare and suggestive form of capsular cataract. Capsular cataract did not often assume so compact form, but was usually observed back of the pupil as a very thin gauze, so thin as only to partially obstruct vision and not always detected, except by observers knowing what they were trying to discover. These gauze-like capsular cataracts may appear after operation for cataract by all the different methods, and are not confined to operations by depression, or with the view of obtaining absorption or solution of the lens mass. This specimen was thick and firm, circular in outline of just sufficient size to close or obstruct the pupil, and was attached quite firmly to the posterior margin of the pupil, preventing all motion, and completely obstructing all vision. It was extracted after separating its attachments by the usual method of extraction of cataracts, and the results had proved satisfactory. He also exhibited some specimens of glandular tumors selected as representative ones from forty-three similar growths, removed from the neck of a little girl 11 years old. Combined, the tumor filled the neck and supra-clavicular region to the point of the shoulder, and consisted of an aggregation of Glandular tumors. He did not regard them as enlarged or distended glands, but as a distinct form of new growth. They were surrounded by a cyst, and thus more or less distinctly separated from each other. Some of them rested behind and beneath the clavicle so as to rest upon the pleura, as near as could be judged by appearances and careful examination. The operation was followed by complete recovery, no unfavorable symptoms appearing at any time, reasonably attributable to it.

On account of the lateness of the hour he would defer description of a tumor recently removed from the carotid region, and requiring ligature of common carotid, and both internal and external carotid, before hemorrhage could be arrested. This case was remarkable in many respects, and required more time to describe it, and he would not longer tresspass upon the time of the association.

Dr. HARTWIG presented a case for diagnosis, the patient, a young man, having a cystic tumor situated at the upper and inner angle of the right orbit.

After the usual routine of business, the meeting adjourned.

L. HOWE, Sec'y, pro tem.

